# Effects of Feed Supplemented with Fermented Pine Needles (*Pinus ponderosa*) on Carcass Quality, Meat Quality, and Antioxidant Capacity of Growing–Finishing Pigs

**DOI:** 10.3390/foods14122046

**Published:** 2025-06-10

**Authors:** Wenfeng Ma, Zhuo Ma, Pei Mao, Xiaoli Zhang, Xiaohong Wu, Mengmeng Gao, Qiujue Wu

**Affiliations:** 1 College of Animal Science and Technology, Henan University of Science and Technology, Luoyang 471023, China; a113boy@163.com (W.M.); maop17@163.com (P.M.); zxl18338890208@163.com (X.Z.); lemonwxh@sian.com (X.W.); 13383955897@163.com (M.G.); 2 Veterinary Medicine and Engineering, Nanyang Vocational College of Agriculture, Nangyang 473000, China; hdzwqj1@163.com

**Keywords:** fermented pine needles, carcass quality, meat quality, antioxidant capacity, finishing pig

## Abstract

The purpose of this study was to investigate the effects of fermented pine needles on the carcass traits, meat quality, and antioxidant capacity of finishing pigs. In total, 80 Duroc × (Landrace × Large white) crossbred pigs of approximately 4 months of age, with an initial body weight of 60.5 ± 2.5 kg, were randomly assigned to four experimental treatments, which were then denoted as the control treatment (basal diet), the fermented pine needle (FR) 1 treatment, the FR2 treatment, and the FR3 treatment (the pigs were fed the basal diet supplemented with 1.0, 2.0, and 3.0% fermented pine needles, respectively) for 55 d. The obtained results showed that, compared with the CON group, the fermented pine needle treatments increased the lean meat percentage, total antioxidative capacity, and superoxide dismutase activity in the serum and *longissimus dorsi* muscle. In addition, the treatments increased the mRNA expression levels of *SOD1*, catalase, and *Nrf2* in the muscle and decreased the malondialdehyde activity in the serum and *longissimus dorsi* muscle and the *Keap1* mRNA expression level. Compared with the CON and FR1 treatment, the FR2 and FR3 treatments increased springiness, serum *GSH-Px* activity, and *longissimus dorsi* muscle CAT activity, and decreased hardness, chewiness, gumminess, and cohesiveness. Moreover, compared with the CON treatment and other fermented pine needle treatments, the FR2 treatment not only significantly elevated the carcass weight, dressing percentage, pH_24h_, a* value (redness), and marbling scores of the finishing pigs, but also remarkably reduced the L* value (lightness), b* value (yellowness), and shear force in the meat quality. In conclusion, the experiment indicated that the addition of fermented pine needles to the diet has no negative impact on the carcass characteristics of finishing pigs and could improve the tenderness and freshness of the meat, as evidenced by the modified antioxidant enzyme activity and mRNA expression levels of antioxidant genes in the muscles of finishing pigs.

## 1. Introduction

Finishing pigs play a pivotal role in China’s production of high-quality pork, effectively meeting the rapidly growing demand for affordable pork [1]. Consumers not only have an increasingly strong preference for finishing pigs and their meat products, but also now attach greater importance to the quality and flavor of pork. Various quality aspects of pork, including meat color, shear force, fat content, and marbling scores, significantly influence consumer purchasing decisions. Consequently, examining the effects of the pigs’ diet on meat quality is of great significance in pork production, offering valuable insights to enhance industry practices and better meet market demands. Over recent decades, numerous studies have demonstrated that meat quality is closely associated with meat color, which serves as a key indicator of freshness and wholesomeness [2,3,4]. The color of meat depends largely on the pH levels, myoglobin and unsaturated fatty acid content, and oxidation processes, all of which affect its marketability and consumer acceptance [5,6]. For this reason, nutritionists and farmers are increasingly incorporating bioactive compounds and herbal additives into pig diets, as these substances are known to have fewer side effects and possess antioxidant properties that contribute to improving the quality of the pork and meeting consumer expectations for healthier meat.

Pine needle (*Pinus* spp.), a traditional herbal medicine, contains diverse phytochemical compounds, such as amino acids (glutamic acid, lysine, phenylalanine, and leucine), phenolics, cinnamic acids, terpenoids, carboxylic acids, fatty acids, flavonoids, and polysaccharides [7]. Owing to these bioactive compounds, pine needles exhibit strong anti-inflammatory, antioxidant, and antimicrobial properties, leading to their widespread use as feed additives [8,9]. For example, the addition of these bioactive compounds into animal feed has been shown to promote free radical scavenging, reduce lipid peroxidation, and enhance endogenous antioxidant enzyme activity, thereby improving meat quality traits such as tenderness, water-holding capacity, and shelf life in finishing pigs [10], poultry [11,12], lambs [13], and cattle [14]. However, some researchers have reported that pine needles contain high levels of condensed tannins, lignocellulose, phenols, and phytates [15], which impair digestibility, reduce feed palatability, and may inhibit nutrient absorption and diet selection [16]. To mitigate these limitations, fermentation with probiotics has emerged as a critical pre-treatment method used to degrade lignocellulose, hydrolyze tannins, enhance the bioavailability of nutrients, and improve antioxidant capacity [7,17,18].

Fermented pine needles have been utilized as a functional animal feed additive due to their various biological properties [7,17]. Studies have reported that fermented pine needles can decrease blood lipid levels and regulate gastrointestinal motility in rats [19] and improve antioxidant capacity in broilers [20]. However, fewer studies have examined the effects of fermented pine needles in pigs, especially in terms of the carcass quality, meat quality, and antioxidant capacity of finishing pigs. Therefore, this study investigated the effects of *Aspergillus niger*-fermented pine needles as a feed additive on these qualities in growing–finishing pigs.

## 2. Materials and Methods

### 2.1. Production of Aspergillus niger-Fermented Pine Needles

The pine needles used in this study were fermented using *Aspergillus niger*. The collection and fermentation of fresh pine needles and the purchase and culturing of *Aspergillus niger* were carried out as described by Wu et al. (2015) [20]. The fermentation medium consisted of a solid mixture of pine needles, soybean meal, wheat bran, and corn cob, along with nutritive salts. After inoculation with 1% *Aspergillus niger* seed, it was fermented at 25 to 30 °C for 48 h. The fermented sample was then packed, sealed, and stored at 24 °C, followed by drying at 30 to 40 °C for 6 days to reach approximately 900 g/kg dry matter (DM) and ground through a 0.5 mm sieve. The changes in selected nutrients and compounds in the pine needles before and after fermentation are described in Wu et al. (2015) [20].

### 2.2. Animals, Diets, and Experimental Design

A single-factor randomized trial was conducted. A total of eighty Duroc × (Landrace × Large white) crossbred pigs of approximately 4 months of age, with an initial body weight of 60.5 ± 2.5 kg, were randomly assigned to 4 experimental treatments according to sex, with 5 replicates in each treatment and 4 pigs in each replicate (two males and two females). The experimental treatments were as follows: the control treatment (pigs fed the basal diet), the fermented pine needle 1 treatment, the fermented pine needle 2 treatment, and the fermented pine needle 3 treatment (pigs fed the basal diet supplemented with 1.0, 2.0, and 3.0% fermented pine needles (FR), respectively). The pre-test period lasted for 7 days, and the experimental period lasted for 48 days. The basal diets were formulated according to the NRC (2012) [21] guidelines for meeting the nutrient requirements of finishing pigs, as detailed in Table 1. Fresh water and feed were available ad libitum.

The pigs were housed in a controlled environment containing 40 pens (2.2 m × 3.5 m). The room was equipped with a temperature-controlled system to maintain temperatures ranging from 23 to 28 °C. The fermented pine needles were added on top of the basal diet. During the experimental period, the pigs were fed two times daily, at 7:00 and 17:00. Meanwhile, the experimental design and procedures were approved by the Animal Care and Use Committee of Henan Science and Technology, following the requirements of the Regulations for the Administration of Affairs Concerning Experimental Animals of China.

### 2.3. Sample Collection

At the end of the experiment, the final live weight was recorded after a period of fasting for 24 h. The next day, 10 mL blood samples were obtained via anterior vena cava puncture and collected in a non-anticoagulant tube. Serum was collected after centrifugation at 3500 r/min (4 °C) for 15 min. The resulting serum samples were stored at −20 °C until lab analysis. After the blood collection, 10 pigs (2 pigs/pen, 5 pens/treatment) were transported (2.5 h) to the slaughterhouse and slaughtered.

Samples of the *longissimus dorsi* muscle (LDM) were collected between the 8th and 10th ribs of the left-side carcass and chilled at 4 °C for further analyses of the meat quality, texture profile, antioxidant enzyme activity, and gene expression. Within 20 min of slaughter, the samples for RNA extraction were promptly removed from the left-side LDM, put into RNase-free tubes, immediately frozen in liquid nitrogen, and then stored at −80 °C until analysis.

### 2.4. Analysis of Carcass Traits

The live weight was measured before slaughtering. The pigs were electrically stunned and slaughtered by exsanguination. The hair was removed; the head, hoof, tail, and internal organs were removed, and the plate oil was retained and weighed. The carcasses were scalded and eviscerated according to GB 12694-2016 (2016) [22]. Following slaughter, the hot carcass weight of each pig was recorded and used to calculate the dressing weight and dressing percentage. The fat depths opposite the first rib, last rib, and last lumbar vertebra were measured to calculate the average backfat thickness, and the muscle thickness at the penultimate 3–4 ribs was recorded and used to calculate the lean meat percentage. The loin eye area was measured at the level of the last rib. Other slaughter characteristic parameters included bone weight, skin weight, and fat weight, and these were used to calculate other carcass parameters.

### 2.5. Measurement of Meat Quality

The evaluation of meat quality was conducted following the established protocols of Beltrán and Roncalés (2000) [23]. The samples were defrosted, and the LDMs were used to measure shear force using a Warner–Bratzler shear (SALTE R, G-R Elec. Mfg. Co. Collins Lane, Manhattan, KS, USA). The measurements were conducted and recorded in accordance with the methodology described by Gurunathan et al. (2022) [24]. Specifically, the meat samples were analyzed 72 h postmortem. For all the samples, the testing parameters were set as follows: a pre-test speed of 2.5 mm s^−1^, a test speed of 2.5 mm s^−1^, and a post-test speed of 10 mm s^−1^. Meat color (brightness, L*; redness, a*; yellowness, b*) was measured 24 h after slaughter on an LDM sample using a colorimeter (NRl0QC, 3nh, Shenzhen, China) standardized with a lightness (L*) of 96.16, redness (a*) of 0.10, and yellowness (b*) of 1.90. The pH values of the meat at 45 min and 24 h post-slaughter were determined from LDM obtained from the left-side carcass using a calibrated pH meter (pH-STAR, SFK-Technology, Copenhagen, Denmark). Cooking loss: the samples were placed in a sealed bag, heated in a water bath at 80 °C for 30 min, and cooled at room temperature for 10 min; then, calculations were made by measuring the weight change of the muscle samples before and after cooking, starting from 45 min post-slaughter. The following method was used to determine the drip loss percentage: approximately 45 min postmortem, a cuboid (5 cm × 3 cm × 2.5 cm) weighing about 30 g was manually trimmed from the LDM and weighed. This sample was then suspended in an inflated plastic bag at 2~4 °C and weighed after 24 h. Drip loss was quantified as the percentage of weight change. The marbling scores (scale of 1 to 5 corresponds with low to high) of the LDM were determined by a trained observer using published visual standards (NPPC, 2000) [25].

### 2.6. Texture Profile Analysis

Textural characteristics were assessed by cutting the cooled meat into 2.0 cm × 2.0 cm × 1.5 cm pieces along the muscle fibers. These samples were then analyzed using a TMS-Pro texture analyzer manufactured by the Food Technology Corporation in Sterling, VA, USA. The probe employed was a flat-bottomed cylindrical P/6 probe with a diameter of 6 mm. The force-sensing element had a measuring range of 25 N. The probe ascended to a height of 15 mm above the surface of the sample. The compression deformation was set at 40%. The pre-test speed was 60 mm/min, the testing speed was 60 mm/min, and the post-test speed was also 60 mm/min. The time interval was 5 s.

### 2.7. Serum and Longissimus Dorsi Muscle Antioxidant Enzyme Activity Analysis

Total RNA was extracted from the LDM using TRIzol reagent (TaKaRa, Dalian, China), followed by purification using RNAeasy spin columns (QIAGEN, Inc., Valencia, CA, USA) according to the manufacturer’s recommendations. Then, a total of 1 μg of total RNA was reverse-transcribed using a PrimeScript Reagent Kit (TaKaRa, Dalian, China) for real-time PCR, and the *GAPDH* and target gene expressions were detected with a CFX connect real-time PCR detection system (CFX-96, Bio-Rad, Hercules, CA, USA) using SYBR green mixture (Q711-02, Vazyme, Nanjing, China) according to the manufacturer’s recommendations. All the PCR reactions were performed under optimized conditions with a range of primer efficiencies. The quality and concentration of total RNA were determined using agarose electrophoresis and a spectrophotometer (Coulter, DU800, Fullerton, CA, USA), respectively. The primer specificities were verified and are listed in Table 2. The *GAPDH* was chosen as the endogenous control gene. The gene amplification efficiencies were calculated according to the specific gene standard curves generated from 10-fold serial dilutions. The 2-ΔΔCT method was used to calculate the expression results.

### 2.8. Statistical Analysis

The statistical analyses of all the experimental data were conducted using SPSS for Windows (version 26.0, SPSS Inc., Chicago, IL, USA). Differences among groups were determined using one-way ANOVA followed by Tukey’s multiple-range tests. The results were expressed as the mean ± standard error of the mean (SEM). A significance level of *p* < 0.05 was considered indicative of a significant difference.

## 3. Results

### 3.1. Carcass Quality

The effects of the inclusion of fermented pine needles on the carcass quality of the finishing pigs are shown in Table 3. No significant differences were found for the body weight before slaughter, dressed weight, loin eye area, backfat thickness, fat percentage, skin percentage, bone percentage, and plate oil percentage among the treatments, but the lean meat percentage was significantly higher in the FR1, FR2, and FR3 treatments compared to the CON treatment in the finishing pigs. The carcass weight and dressing percentage were significantly higher in the FR2 treatment compared to the CON treatment, but no differences in the carcass weight or dressing percentage were observed among the CON, FR1, and FR3 treatments.

### 3.2. Meat Traits

The effects of the inclusion of fermented pine needles on the meat traits of finishing pigs are shown in Table 4. The pH_24h_, a*, and marbling scores were significantly increased in the FR2 treatments compared to the CON treatment, but no differences in the pH_24h_, a*, or marbling scores were observed among the CON, FR1, and FR3 treatments. Moreover, the L*, b*, and shear force of the meat were significantly lower in the FR2 treatments compared to the CON treatment, but no differences in the L*, b*, or shear force were observed among the CON, FR1, and FR3 treatments. No significant differences were observed for dripping loss, cooking loss, or pH_45min_ among the treatments (*p* > 0.05).

### 3.3. Muscles Texture Characteristics

The effects of the inclusion of fermented pine needles on the muscle texture characteristics of the finishing pigs are shown in Table 5. The springiness was significantly higher in the FR2 and FR3 treatments than in the CON and FR1 treatments, but no differences in springiness were observed between the CON and FR1 treatments or between FR2 and FR3. The hardness, chewiness, gumminess, and cohesiveness were significantly lower in the FR2 and FR3 treatments than in the CON and FR1 treatments, but no differences were found between the CON and FR1 or the FR2 and FR3 treatments.

### 3.4. Serum and Longissimus Dorsi Muscle Antioxidant Enzyme Activity

The effects of the inclusion of fermented pine needles on the serum and *longissimus dorsi* muscle antioxidant enzymes of the finishing pigs are shown in Table 6. The MDA activity in the serum and *longissimus dorsi* muscle was significantly lower in the FR1, FR2, and FR3 treatments compared to the CON treatment in the finishing pigs, but no differences in MDA activity were observed among the FR1, FR2, and FR3 treatments (*p* > 0.05). The T-AOC and SOD activity in the serum and *longissimus dorsi* muscle were significantly higher in the FR1, FR2, and FR3 treatments compared to the CON treatment (*p* < 0.05), but no differences in the T-AOC, SOD, or CAT activity were observed among the FR1, FR2, and FR3 treatments (*p* > 0.05). There were no significant differences in serum CAT activity or *longissimus dorsi* muscle *GSH-Px* activity among the FR1, FR2, FR3, and CON treatments (*p* > 0.05). The serum *GSH-Px* activity and *longissimus dorsi* muscle CAT activity were significantly higher in the FR2 and FR3 treatments compared to the CON and FR1 treatments (*p* < 0.05), but no differences were observed between the FR2 and FR3 or the CON and FR1 treatments.

### 3.5. The mRNA Expression of Antioxidant Genes in the Muscle

**Figure 1:**
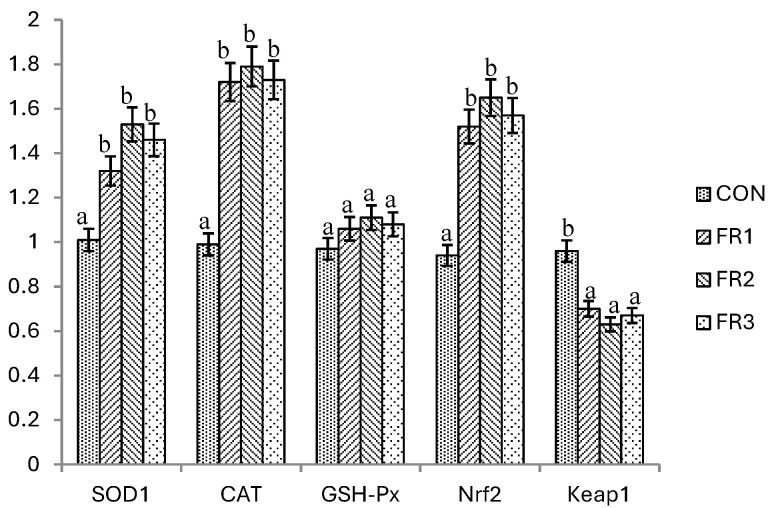
Effects of the inclusion of fermented pine needles on the mRNA expression of antioxidant genes in the muscle of finishing pigs. The letters a and b in the figure indicate significant differences.

Effects of the inclusion of fermented pine needles on the mRNA expression of antioxidant genes in the muscles of the finishing pigs are shown in Figure 1. The mRNA expression levels of *SOD1*, CAT, and *Nrf2* in muscle were significantly higher in the FR1, FR2, and FR3 treatments compared to the CON treatment in the finishing pigs. No significant differences were observed in terms of the *SOD1*, CAT, and *Nrf2* mRNA expression levels among the fermented pine needle treatments. The mRNA expression level of *Keap1* in muscles was significantly lower in the FR1, FR2, and FR3 treatments compared to the CON treatment, but no differences were observed among the FR1, FR2, and FR3 treatments. Moreover, no significant differences were found in the *GSH-Px* mRNA expression levels of the jejunal muscle between the CON treatment and fermented pine needle treatments.

## 4. Discussion

### 4.1. Effects of the Inclusion of Fermented Pine Needles on the Carcass and Meat Traits of Finishing Pigs

The slaughter characteristics of carcasses (dressed weight, carcass weight, loin eye area, etc.) and meat traits (marble score, meat color, shear force, etc.) are the main indicators for evaluating meat quality in livestock and poultry [26,27]. Previous studies have found that the supplementation of pine needles improves the meat traits and meat color stability via antioxidant activity in the muscles of broilers [9,28] and in smoked bacon [29]. Similar results were also reported by Seo et al. (2020) [28], Kim et al. (2012) [30], and Kim et al. (2014) [31], who evaluated the meat quality of broilers fed pine needles in their feed. The results of our present experiment confirmed that adding fermented pine needles into feed also affects the meat traits of growing–finishing pigs. A reason for this may be that the addition of fermented pine needles leads to an increase in the absorption of amino acids and other nutrients in the muscles of the animals [28]. Moreover, we also found that adding fermented pine needles to the diet can improve the shear force and muscle texture characteristics of the meat from growing–finishing pigs. The experimental results are inconsistent with the conclusions of the studies mentioned above [28,30,31] and may reflect the type of muscle fiber in pigs and chickens.

In our evaluation of meat color, it was observed that a concentration of 2.0% fermented pine needles had an effect on the L* and a* values, demonstrating a potent antioxidant effect. The color parameters in fresh pork are presumed to be linked directly with myoglobin content, as previous studies have documented [32]. The total myoglobin content in the meat was affected by the dietary fermented pine needle treatment [20]. Antioxidant activity is a well-recognized biological property, this activity is predominantly attributed to its flavonoid and phenolic compounds in pine needles [31,33,34]. These compounds not only have the ability to act as antioxidants by scavenging free radicals and delaying metmyoglobin formation in meat, but are also able to chelate transition metals from iron ions [35,36]. Based on the accumulated evidence from these studies, it can be concluded that the following factors predominantly contribute to the variations in meat color: pH, metmyoglobin formation, the oxidation process, and the heme pigment concentration [30,37,38]. As mentioned above, natural additives with an antioxidant function may be beneficial in maintaining meat quality.

Similarly, dripping loss and cooking loss are very important meat trait parameters and reflect the juiciness of the meat [39,40]. In this study, the fermented pine needle treatments had no effect on the dripping loss and cooking loss of the meat from the finishing pigs. These results were in agreement with the results for backfat thickness, fat percentage, and plate oil percentage. In general, a higher intramuscular fat content is associated with greater meat juiciness, as previously reported [41]. Moreover, an increased meat fat content improves the water-holding capacity of the meat and reduces drip loss [42]. However, in the treatments with fermented pine needles, the pH value of the meat at 24 h was higher, and the shear force was lower. This is inconsistent with the fat content. This finding indicates that the addition of different amounts of fermented pine needles can improve the contents of protein, glycogen, and lactic acid in the muscles, as well as the structure and condition of the muscle fibers. The pH value is a physical indicator of muscle acidity and alkalinity and is related to the degradation of glycogen and the release of lactic acid [27]. These observed effects can likely be attributed to the bioactive compounds present in fermented pine needles, including phenolics, terpenoids, flavonoids, and polysaccharides. These compounds can inhibit muscle glycolysis by down-regulating glycolysis-related enzymes [43,44], reducing muscle lactic acid accumulation after slaughter, improving the pH value, and slowing down the decline in the rate of pH [45].

The marble scores were also analyzed. As expected, the marble scores were significantly higher in the FR2 treatment than those of the CON and other fermented pine needle treatments, indicating that supplementation with fermented pine needles can improve the juiciness and tenderness of muscles. This may be because fermented pine needles are rich in antioxidants and characteristic bioactive compounds [20]. Some studies reported that self-fermented pine needles contain several essential oils, lipids, carbohydrates, and terpenoids, including 1,8-cineole, and exhibit high antioxidant activity [20,46]. In the present experiment, we evaluated the effect of fermented pine needles on antioxidant activity in pigs, which may be attributed to the induced antioxidative effect of the fermented pine needles on the pig muscle and serum. On the other hand, lipid oxidation can alter the chemical properties of heme and cause myoglobin oxidation, resulting in a change in meat color [32]. Antioxidants can inhibit the conversion of myoglobin in meat to metmyoglobin, protecting mutton from discoloration [47], thus affecting the grading and color of meat. These findings indicate that the carcass and meat trait indexes of the meat from pigs treated with fermented pine needles are superior to those of the control group.

### 4.2. Effects of the Inclusion of Fermented Pine Needles on the Muscle Texture Characteristics of Finishing Pigs

Textural parameters such as hardness, chewiness, and springiness are related to the tenderness and palatability of the meat [48]. This study determined the texture traits (hardness, springiness, chewiness, gumminess, and cohesiveness) of meat from pigs fed different fermented pine needle concentrations. The analysis of the test results shown in Table 5 found no significant effect of the fermented pine needles on the springiness of the meat, but the meat from the pigs fed 2.0% and 3.0% fermented pine needles had lower hardness, chewiness, gumminess, and cohesiveness compared to the meat from the other groups. These results indicated that the tissue structure and fiber condition of the *longissimus dorsi* muscles exhibited different changes according to the concentration of fermented pine needles consumed. No previous study has reported the effects of fermented pine needle consumption on the hardness, springiness, chewiness, gumminess, or cohesiveness of meat from pigs. However, our findings conclude that adding fermented pine needles to the diet can improve some textural characteristics, to a certain extent, and positively affect the quality of pork. These effects may be achieved by regulating muscle protein stability and antioxidant activity, as well as the tissue structure.

### 4.3. Effects of the Inclusion of Fermented Pine Needles on the Antioxidant Enzyme Activity and the mRNA Expression of Antioxidant Genes of Finishing Pigs

To confirm the above hypothesis, we examined the antioxidant parameters of the serum and muscle and determined the mRNA expression of antioxidant genes in the muscle. We observed that the fermented pine needle diet group exhibited higher antioxidant enzyme (T-AOC, SOD, *GSH-Px*, CAT) activity, mRNA levels of antioxidant genes (*SOD1*, *CAT*, and *Nrf2*), and lower MDA activity and *Keap1* mRNA levels than the control group. These antioxidant enzymes and genes play important roles in balancing body oxidation and antioxidation. The findings regarding the activity of antioxidant enzymes in our current study are consistent with those reported by Wu et al. [20]. Moreover, these results are all consistent with the higher pH, meat color a* value, lower shear force, hardness, chewiness, gumminess, and cohesiveness values. These results suggest that fermented pine needle supplementation in finishing pigs is beneficial for protecting muscle tissues. This benefit could be an effect of dietary biological substances (total polysaccharides, phenolics, and flavonoids), which may also be explained by a more general mechanism of reducing cellular antioxidant consumption by reducing the burden of the enzymatic and nonenzymatic antioxidative system [20]. It is evident that further research is essential to fully elucidate the biological functions and underlying mechanisms of fermented pine needles in pig nutrition and production.

## 5. Conclusions

In summary, the inclusion of fermented pine needles in the diet of finishing pigs resulted in superior meat traits (such as the tenderness and freshness of the meat), as evidenced by the lower shear force, hardness, chewiness, gumminess, and cohesiveness; higher pH_24h_; meat color a* value; marbling scores; and oxidative enzyme activity. The underlying mechanisms may be associated with the higher antioxidant enzyme activity and mRNA expression levels of antioxidant genes in the muscles of finishing pigs. Further research is required to identify an optimal level of dietary fermented pine needles that has the best effects on pig carcass performance and improves meat traits and to reveal the underlying molecular and physiological mechanisms that govern these beneficial effects.

## Figures and Tables

**Table 1:** Composition and nutrient levels of the diet (DM basis), %.

Ingredients	Content (%)	Nutrient Level	Content (%)
Corn	40.50	Calculated chemical composition	
Expanded corn	12.30	Digestible energy (MJ/kg)	13.48
Wheat bran	3.50	Crude protein	19.89
Soybean meal	2.00	Ca	0.85
Expanded soybean	9.50	Total phosphorus	0.55
Bean	17.00	Available phosphorus	0.50
Fish meal	4.50	D-lysine	1.28
Whey powder	2.00	D-methionine	0.45
Sucrose	2.50	D-Met+D-Cys	0.69
CaCO_3_	0.75	D-threonine	0.80
CaHPO_4_	1.70	D-tryptophan	0.25
NaHCO_3_	0.50		
L-lysine-HCl	0.35		
DL-methionine	0.05		
L-threonine	0.05		
Soda	0.50		
Limestone	0.90		
NaCl	0.35		
Vitamin–mineral premix ^1^	0.90		
Choline chloride	0.15		
Total	100.00		

^1^ The premix provided the following per kg of the diets: Vitamin A 2500 IU; Vitamin D 250 IU; Vitamin E 18 IU; Vitamin K_3_ 0.5 mg; Vitamin B_1_ 0.50 mg; Vitamin B_2_ 5.00 mg; Vitamin B_5_ 12 mg; Vitamin B_6_ 2.00 mg; Vitamin B_12_ 0.02 mg; Folic acid, 0.3 mg; Nicotinic acid 20 mg; Pantothenic acid 14 mg; biotin 0.08 mg; Cu (CuSO_4_·5H_2_O) 100 mg; Fe (FeSO_4_·H_2_O) 75 mg; Mn 30 mg; Zn (ZnSO_4_·H_2_O) 45 mg.

**Table 2:** Primer sequence information used for real-time quantitative PCR.

Gene ^1^	Primer Sequence (5′-3′)	Length (bp)
*GAPDH*	F:ACTCACTTCTACCTTTGATGCT	100
	R:TGTTGCTGTAGCCAAATTCA	
*GSH-Px*	F:GTGACTCACGCAAATGCTCC	125
	R:ATTGAGGCACTGGAGACA	
*SOD1*	F:AGACCTGGGAAGTGTGACTG	102
	R:GTGCGGCCACTAATGGAATG	
*Nrf2*	F:CCAGTCTTCATTGCTCCTAACCA	129
	R:CCTCCCAAACTTGCTCAATATCCT	
*Keap1*	F:CAGCTTCGCGGAGCAGATTGG	129
	R:CTAGCAGTGGCACAAATTGAAGAA	

^1^ GAPDH, glyceraldehyde-3-phosphate dehydrogenase.

**Table 3:** Effects of the inclusion of fermented pine needles on carcass traits of finishing pigs.

Items	Treatments ^1^				*p*-Value ^2^
	CON	FR1	FR2	FR3	
Body weight before slaughter/kg	96.42 ± 3.26	96.58 ± 4.75	99.98 ± 2.43	98.97 ± 2.96	0.213
Dressed weight/kg	89.32 ± 2.15	90.15 ± 1.02	91.77 ± 1.33	91.50 ± 1.64	0.125
Carcass weight/kg	69.85 ± 1.08 ^a^	69.88 ± 2.11 ^a^	75.52 ± 1.42 ^b^	71.63 ± 1.19 ^a^	0.031
Dressing percentage/%	72.44 ± 0.94 ^a^	72.35 ± 1.64 ^a^	75.54 ± 0.15 ^b^	72.38 ± 0.31 ^a^	0.034
Loin eye area/cm^2^	37.82 ± 4.12	38.95 ± 3.17	40.75 ± 2.36	40.38 ± 2.68	0.268
Backfat thickness/cm	1.53 ± 0.16	1.63 ± 0.43	1.76 ± 0.33	1.65 ± 0.29	0.394
Lean meat percentage/%	60.39 ± 0.10 ^a^	61.56 ± 0.21 ^b^	62.35 ± 0.19 ^b^	61.69 ± 0.15 ^b^	0.010
Fat percentage/%	4.35 ± 0.31	4.32 ± 0.23	4.29 ± 0.15	4.30 ± 0.24	0.359
Skin percentage/%	3.15 ± 0.20	3.08 ± 0.27	2.95 ± 0.31	3.04 ± 0.26	0.651
Bone percentage/%	8.86 ± 0.41	8.69 ± 0.35	8.49 ± 0.24	8.53 ± 0.43	0.524
Plate oil percentage/%	0.81 ± 0.08	0.74 ± 0.06	0.77 ± 0.12	0.73 ± 0.11	0.567

Note: ^1^ Control = Basal diet; FR1 = Basal diet supplemented with 1.0% fermented pine needles; FR2 = Basal diet supplemented with 2.0% fermented pine needles; FR3 = Basal diet supplemented with 3.0% fermented pine needles. ^2,a,b^ Means within the same row that do not share a common superscript are significantly different (*p* < 0.05); *n* = 10.

**Table 4:** Effects of the inclusion of fermented pine needles on meat traits of finishing pigs.

Items	Treatments ^1^				*p*-Value ^2^
	CON	FR1	FR2	FR3	
pH_45min_	6.40 ± 0.10	6.43 ± 0.14	6.53 ± 0.16	6.48 ± 0.11	0.395
pH_24h_	5.40 ± 0.01 ^a^	5.42 ± 0.05 ^a^	5.56 ± 0.03 ^b^	5.43 ± 0.02 ^a^	0.001
L*	38.24 ± 0.53 ^b^	37.69 ± 0.61 ^b^	34.05 ± 0.28 ^a^	36.85 ± 0.34 ^b^	0.002
a*	3.81 ± 0.10 ^a^	4.12 ± 0.14 ^a^	4.48 ± 0.09 ^b^	4.23 ± 0.11 ^a^	0.034
b*	5.01 ± 0.08 ^b^	4.86 ± 0.11 ^b^	4.51 ± 0.12 ^a^	4.76 ± 0.10 ^b^	0.041
Dripping loss/%	3.28 ± 0.20	3.26 ± 0.15	3.11 ± 0.13	3.08 ± 0.12	0.601
Cooking loss/%	24.46 ± 1.35	23.67 ± 2.37	22.58 ± 3.51	23.04 ± 1.94	0.412
Shear force/N	45.25 ± 4.35 ^b^	44.60 ± 4.82 ^b^	40.43 ± 5.21 ^a^	43.65 ± 4.26 ^b^	0.012
Marbling scores	2.58 ± 0.16 ^a^	2.69 ± 0.21 ^a^	3.06 ± 0.23 ^b^	2.72 ± 0.19 ^a^	0.029

Note: ^1^ Control = Basal diet; FR1 = Basal diet supplemented with 1.0% fermented pine needles; FR2 = Basal diet supplemented with 2.0% fermented pine needles; FR3 = Basal diet supplemented with 3.0% fermented pine needles. ^2,a,b^ Means within the same row that do not share a common superscript are significantly different (*p* < 0.05); *n* = 10.

**Table 5:** Effects of the inclusion of fermented pine needles on muscle texture characteristics of finishing pigs.

Items	Treatments ^1^				*p*-Value ^2^
	CON	FR1	FR2	FR3	
Hardness/N	37.81 ± 2.25 ^b^	34.50 ± 1.28 ^b^	26.24 ± 1.39 ^a^	28.78 ± 1.64 ^a^	0.029
Springiness/mm	3.91 ± 0.21 ^a^	4.32 ± 0.16 ^a^	5.31 ± 0.28 ^b^	4.96 ± 0.14 ^b^	0.041
Chewiness/N	71.78 ± 1.92 ^b^	68.33 ± 2.04 ^b^	52.14 ± 3.01 ^a^	58.39 ± 2.73 ^a^	0.038
Gumminess/N	20.13 ± 1.01 ^b^	19.05 ± 2.11 ^b^	12.53 ± 2.04 ^a^	13.29 ± 2.35 ^a^	0.024
Cohesiveness	0.51 ± 0.03 ^b^	0.49 ± 0.06 ^b^	0.41 ± 0.04 ^a^	0.43 ± 0.02 ^a^	0.012

Note: ^1^ Control = Basal diet; FR1 = Basal diet supplemented with 1.0% fermented pine needles; FR2 = Basal diet supplemented with 2.0% fermented pine needles; FR3 = Basal diet supplemented with 3.0% fermented pine needles. ^2,a,b^ Means within the same row that do not share a common superscript are significantly different (*p* < 0.05); *n* = 10.

**Table 6:** Effects of the inclusion of fermented pine needles on serum and *longissimus dorsi* muscle antioxidant enzymes of finishing pigs.

Items		Treatments ^1^				*p*-Value ^2^
		CON	FR1	FR2	FR3	
Serum	T-AOC (u/mL)	0.33 ± 0.01 ^a^	0.37 ± 0.02 ^b^	0.39 ± 0.02 ^b^	0.38 ± 0.03 ^b^	0.013
	SOD (u/mL)	63.04 ± 0.52 ^a^	68.31 ± 0.41 ^b^	69.35 ± 0.20 ^b^	68.48 ± 0.25 ^b^	0.024
	CAT (u/mL)	6.25 ± 0.32	6.89 ± 0.19	7.15 ± 0.26	7.06 ± 0.41	0.053
	*GSH-Px* (u/mL)	706.35 ± 24.15 ^a^	719.82 ± 20.53 ^a^	775.68 ± 22.61 ^b^	763.01 ± 25.18 ^b^	0.038
	MDA (nmol/mL)	5.76 ± 0.19 ^b^	4.31 ± 0.12 ^a^	3.82 ± 0.16 ^a^	4.05 ± 0.17 ^a^	0.003
*Longissimus dorsi* muscle	T-AOC (U/mg prot)	0.13 ± 0.01 ^a^	0.18 ± 0.02 ^b^	0.20 ± 0.03 ^b^	0.19 ± 0.01 ^b^	0.012
	SOD (U/mg prot)	7.99 ± 0.21 ^a^	9.17 ± 0.24 ^b^	9.88 ± 0.30 ^b^	9.53 ± 0.22 ^b^	0.011
	CAT (U/mg prot)	1.79 ± 0.31 ^a^	2.21 ± 0.40 ^a^	3.24 ± 0.38 ^b^	3.11 ± 0.29 ^b^	0.037
	*GSH-Px* (U/mg prot)	1.54 ± 0.11 ^a^	1.73 ± 0.14 ^a^	1.81 ± 0.12 ^a^	1.79 ± 0.15 ^a^	0.020
	MDA (nmol/mg prot)	0.28 ± 0.01 ^b^	0.11 ± 0.02 ^a^	0.09 ± 0.01 ^a^	0.10 ± 0.03 ^a^	0.011

Note: ^1^ Control = Basal diet; FR1 = Basal diet supplemented with 1.0% fermented pine needles; FR2 = Basal diet supplemented with 2.0% fermented pine needles; FR3 = Basal diet supplemented with 3.0% fermented pine needles. ^2,a,b^ Means within the same row that do not share a common superscript are significantly different (*p* < 0.05); *n* = 10.

## Data Availability

The data that support the findings of this study are available from the corresponding author upon reasonable request.
